# The colors of biomass burning aerosols in the atmosphere

**DOI:** 10.1038/srep28267

**Published:** 2016-06-16

**Authors:** Chao Liu, Chul Eddy Chung, Feng Zhang, Yan Yin

**Affiliations:** 1 Collaborative Innovation Center on Forecast and Evaluation of Meteorological Disasters, Nanjing University of Information Science & Technology, Nanjing 210044, China; 2 Key Laboratory for Aerosol-Cloud-Precipitation of China Meteorological Administration, School of Atmospheric Physics, Nanjing University of Information Science and Technology, Nanjing 210044, China; 3 Desert Research Institute, Reno, NV 89512, USA; 4 Key Laboratory of Meteorological Disaster of Ministry of Education, Nanjing University of Information Science & Technology, Nanjing 210044, China

## Abstract

Biomass burning aerosols mainly consist of black carbon (BC) and organic aerosols (OAs), and some of OAs are brown carbon (BrC). This study simulates the colors of BrC, BC and their mixture with scattering OAs in the ambient atmosphere by using a combination of light scattering simulations, a two-stream radiative transfer model and a RGB (Red, Green, Blue) color model. We find that both BCs and tar balls (a class of BrC) appear brownish at small particle sizes and blackish at large sizes. This is because the aerosol absorption Ångström exponent (AAE) largely controls the color and larger particles give smaller AAE values. At realistic size distributions, BCs look more blackish than tar balls, but still exhibit some brown color. However, when the absorptance of aerosol layer at green wavelength becomes larger than approximately 0.8, all biomass burning aerosols look blackish. The colors for mixture of purely scattering and absorptive carbonaceous aerosol layers in the atmosphere are also investigated. We suggest that the brownishness of biomass burning aerosols indicates the amount of BC/BrC as well as the ratio of BC to BrC.

Some aerosol species are named after their chemical composition, such as sulfate and nitrate. On the other hand, black carbon (BC) and brown carbon (BrC) aerosols are carbonaceous aerosols in their chemical composition, and named after their visual color appearance. For instance, BrC, a class of organic aerosol (OA)^1^,^2^, is named as “BrC” because it looks brownish^3^. In particular, some organic aerosols were found to absorb solar radiation more efficiently at shorter wavelengths compared to longer wavelengths^3^,^4^,^5^, and relatively high absorption of shorter visible light leads to more scattering in the red region and gives brown color (instead of black)^3^,^5^. As a result, this absorptive component of OAs is called BrC. Meanwhile, tar balls (TBs) that are widely observed during different biomass burning events in the form of solid spheres are also a class of BrC^6^,^7^,^8^,^9^,^10^,^11^, and show significant uncertainties on their colors, microphysical and optical properties.

In the ambient atmosphere, aerosols generally exist as mixtures of different species, and nobody has observed single species of aerosol alone in the atmosphere. Aerosols from vegetation fires, mostly comprising BC and OAs, display a mixture of black, brown, grey and white colors, and this color variation, from blackish to whitish, has been attributed to the relative ratio of BC to BrC and OA^2^,^12^. This common thinking is an untested hypothesis, and furthermore contradicts a well-known optics theory that absorptive aerosols of any color in the atmosphere become blackish if the layer is thick and dense enough. As shown in Fig. 1a, flaming forest fire smokes, known to emit a lot of BC, actually look quite brownish, and more blackish parts appear related to denser smoke, leaving people with a question of where so-called blackish BC particles are in this fire.

BC and BrC have been accepted to look blackish and brownish^3^, respectively, not because the BC or BrC color was observed in the ambient atmosphere. The color of aerosols has been derived by the color of aerosol material or their solution^3^,^4^, since the material of a specific aerosol species can be isolated, e.g., by dissolution in acetone or in water^4^, or aerosol filter samples^3^. However, the color of aerosols in the atmosphere might differ substantially from that of the aerosol material, because the aerosol absorption and scattering properties are significantly influenced by their size distribution and geometry, which might be destroyed during the dissolution. The color of an aerosol filter sample is not an accurate indicator of the aerosols in the atmosphere, because filters do not well preserve the ambient conditions of aerosol size distribution or shape either.

Smokes in the ambient atmosphere are always complicated mixtures of various aerosol species^2^,^12^, and we do not know what color each aerosol species or mixture produces in the ambient atmosphere. For instance, does BrC actually look brownish if it exists without the interferences of other particles in the atmosphere? How dense should a BrC aerosol layer be to look blackish? Can BC also look brown under some circumstances? These questions create a need to numerically investigate the color of aerosols in their ambient conditions. To our knowledge, we present the first study to simulate the color of aerosols in the atmosphere. We choose biomass burning aerosols as a case study since there is little NO_2_ emission (which gives brownish color and interferes with the color of aerosols), and the aerosols, mainly BC and OA, would determine the color. Thus, in case of biomass burning aerosols, our aerosol color simulation would have direct applications to understand the visual appearance of the smoke.

Figure 1b illustrates the configuration of our simulation. The simulation scheme is designed to mimic realistic ambient condition, in which solar light reaches an observer after passing through an aerosol layer. In this figure, the incident light, i.e. purely white light, is scattered and absorbed by the aerosol layer, and the transmitted light (both direct and diffuse energy), represented by symbol “T” in the figure, is used to calculate and display the color seen by the observer. Randomly distributed spheres in the figure represent aerosol particles in the atmosphere, and their spectral-dependent optical properties determine the relative strengths of the transmitted monochromatic red, green and blue light beams, and, thus, the color.

We simulate the color of biomass burning aerosols in the ambient conditions, and both pure BrC/BC and external mixtures of scattering OA (non-absorptive) and BrC/BC are considered in this study. The light scattering properties of the aerosols are calculated based on geometries of sphere, aggregate or core-shell inhomogeneous sphere, and the transmittances of the red, green and blue light obtained from a simple radiative transfer model are used to represent the RGB triplet (r, g, b) for the color display. This study considers TBs based on observations from three literatures, i.e. namely TB-C^6^, TB-H^7^ and TB-A^8^, and BCs with different geometries are studied, i.e. Lacy-BC, Compact-BC and Coated-BC. These three kinds of TBs have quite different wavelength-dependent optical properties, while, in terms of geometry, all the three are spheres. For BC, we also consider three cases, which are assumed to have the same “material (i.e. refractive indices)” but different geometries. The lacy aggregate, compact aggregate and core-shell sphere are considered to account for BC aerosols at different aging stages and coating effects. The aggregates are numerically described by the fractal aggregates with different fractal dimensions^13^,^14^. Figure 2 shows examples of the particle geometries we consider.

## Results

### Properties of biomass burning aerosols

The refractive index is one of the most fundamental parameters differing aerosol species and their colors, and can significantly help us to understand the aerosol colors. Figure 3 illustrates the refractive indices of the absorptive carbonaceous species at wavelengths between 400 nm and 800 nm. The refractive indices of the TB-C^6^, TB-H^7^ and TB-A^8^ are obtained from the corresponding literatures, and those reported by Chang and Charalampopoulos^15^ are used for the BC. For the TB-C and TB-H, values at only three wavelengths are obtained, and those of the TB-A and BC have much finer spectral resolution. The absorptivity of the four aerosols can be easily understood by comparing their imaginary parts of refractive indices. In the visible wavelengths, the TB-C shows imaginary parts of refractive indices on the order of 10^−3^, whereas values around 0.2 and 0.5 are shown for other TBs and BC, respectively. Furthermore, the imaginary parts of the TB-H are slightly smaller than those of the TB-A, and decreases more rapidly at visible wavelengths as wavelength increases. The spectral variation of the carbonaceous aerosol refractive indices determines their relative optical properties at different wavelengths, and, thus, their color in the atmosphere.

It should be noticed that there are still significant uncertainties on the refractive indices of the TBs and BCs, although they are directly retrieved from observed optical properties^6^,^7^,^8^,^15^. Errors on aerosol refractive indices can be easily introduced by different instruments and retrieval methods, and their microphysical properties such as the size distribution and particle geometry also influence the retrieved results. Thus, this study only focuses on the differences given by different TB species or BCs with different geometries, and the uncertainties on the refractive indices are not discussed.

Table 1 compares the assumed geometries, typical diameters and simulated optical properties (i.e. extinction Ångström exponent (EAE) and absorption Ångström exponent (AAE)) of the six kinds of carbonaceous aerosols. Considering our classification and the imaginary parts of refractive indices, the absorptivity of the aerosols is classified into three types, i.e. weak, moderate and strong. Four geometries, i.e. sphere, lacy aggregate, compact aggregate and core-shell sphere, are illustrated in Fig. 2. For the Lacy- and Compact-BCs, the fractal aggregates with monomer diameter of 30 nm are considered, and the fractal dimensions of 1.8 and 2.8 are used, respectively. Each aggregate in Fig. 2 contains 100 monomers, and the sphere and core of the core-shell sphere in the figure have the same volume as those of the aggregates. For Coated-BC, the coating (sulfate in this study) is assumed to have the same volume as that of the BC core. We use lognormal size distributions for all the carbonaceous aerosol species. The diameter in the table is referred to as the geometric mean values of the lognormal size distribution, and the values close to the typical observed values are used following^6^,^7^,^8^. The geometric standard deviation in the size distribution is fixed to be 1.5, which is standard in aerosol simulations^16^. For homogeneous and core-shell spherical particles, the optical properties can be efficiently obtained by the Lorenz-Mie theory^17^ and its extension^18^, and the optical properties of aggregates are calculated using the multiple-sphere T-matrix method^14^,^19^,^20^. The EAE and AAE values are approximated based on bulk extinctions and absorptions between wavelengths of 436 nm (blue) and 700 nm (red). The EAEs of the TBs vary from 0.6 to 2.6, whereas values from 1.1 to 1.4 are obtained for the BCs. The AAEs we calculated reside in the range of observed values^6^,^7^. For example, the observed AAEs for the TB-C and TB-H are between 2 and 7, and between 2.5 and 3.5, respectively, and our simulation gives values of 4.3 and 2.8. The calculated AAE of the TB-A is quite different from that of the reference^8^, because the value of a single-sized particle is given in their work. The TB-C and TB-H show AAE values larger than 2, and those of three BC aerosols range between 0.9 and 1.2.

### Colors of biomass burning aerosols with single species

Figure 4 illustrates the colors of the six carbonaceous aerosols at different particle sizes (i.e., different geometric mean diameters) and optical depths. We consider aerosol layers with optical depth (at 546 nm) ranging from 0 up to 5. The incident angle is set to be 30°. Figure 4 clearly demonstrates that the color of aerosol layers is highly sensitive to aerosol type, size distribution and optical depth. It is expected that, as the optical depth increases, the color of the aerosol layer becomes darker, because the layer reflects and absorbs more incident energy. With much weaker absorptivity, the TB-C shows brighter color, and is brown at almost any size and optical depth. The TB-H and TB-A also show different brownish colors. The TB-H appears to be brown when its mean diameter and optical depth are less than approximately 0.2 μm and 3, respectively, whereas smaller size and thinner optical depth are required for the TB-A to show brown colors. This is because of that the brownish colors are partially caused by the different absorption at three monochromatic wavelengths, and the AAE generally decreases as particles become larger. The BCs with different geometries show very close colors from white, to grey, and to black as the optical depth increases, and their colors are also less sensitive to the particle mean diameter. The BCs in the form of lacy and compact aggregates show quite similar colors in the atmosphere, which indicates that the collapse of BC aggregates during the aging has little influence on the aerosol color. We notice that the Coated-BC is not always blackish (or grey) and is brownish at small geometric mean diameters and optical depth. Overall, considering the variance of AAE values on the geometric mean diameter, we find that the brown color can be obtained if the AAE of the aerosol and transmittance at 546 nm are approximately larger than 2 and 0.15, respectively.

**Figure 1:**
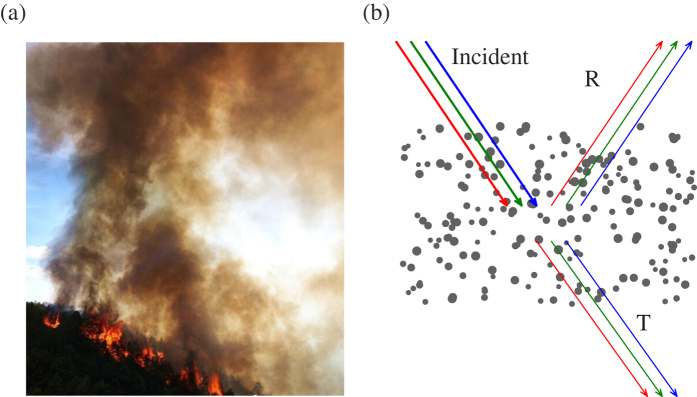
(**a**)A colorful smoke image from a forest fire as occurred in Sichuan province, China, on May 31st, 2014 (from http://www.chinanews.com/tp/hd2011/2014/06-01/355586.shtml), and (**b**) Configuration to simulate colors of ambient biomass burning (carbonaceous) aerosols. The transmittance of the red, green and blue light is used for color display in the RGB color model.

**Figure 2:**
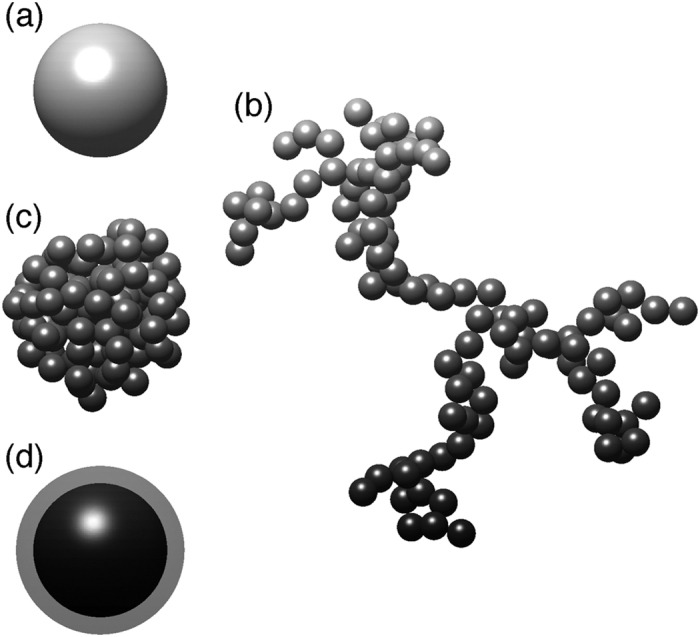
Particle geometries assumed in this study. (**a**) A sphere (for TB-C, TB-H and TB-A), (**b**) A lacy aggregate with a fractal dimension of 1.8 (for Lacy-BC), (**c**) A compact aggregate with a fractal dimension of 2.8 (for Compact-BC), and (**d**) A core-shell sphere (for Coated-BC). Each aggregate in the figure contains 100 monomers. The volumes of carbonaceous materials of the four particles are the same, and the black carbon core and sulfate shell are assumed to have the same volume for the Coated-BC.

**Figure 3:**
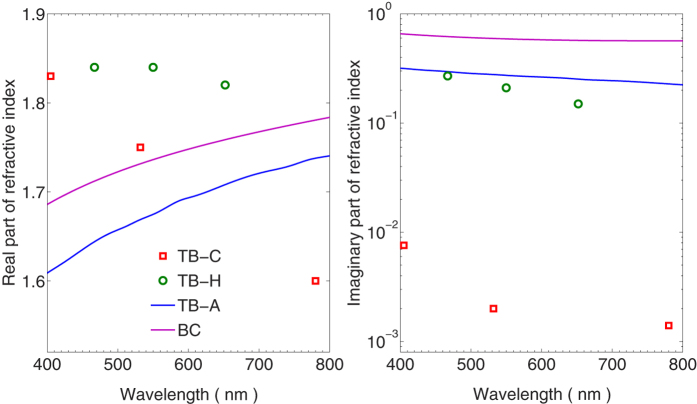
Real part (left) and imaginary part (right) of complex refractive indices for the carbonaceous aerosols as functions of wavelength.

**Figure 4:**
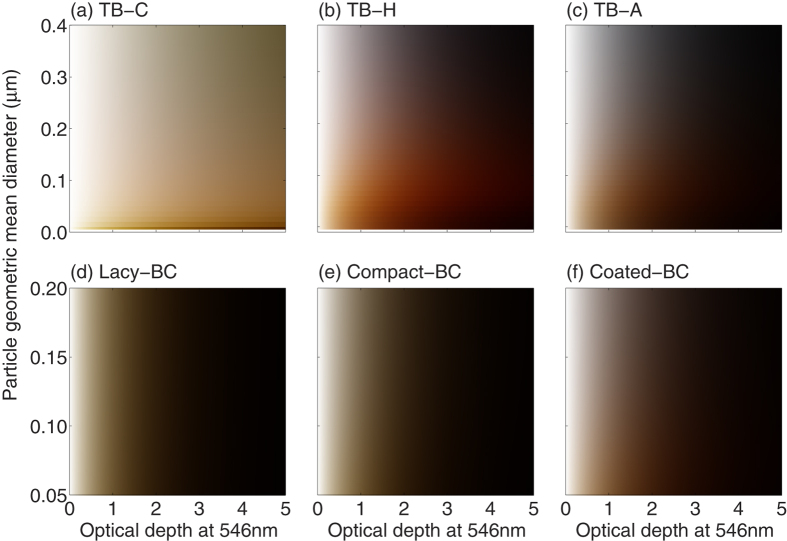
Colors of carbonaceous aerosol layers with different optical depth and particle geometric mean diameters. The optical depth is for the value of the aerosol layer at the green wavelength (546 nm).

**Figure 5:**
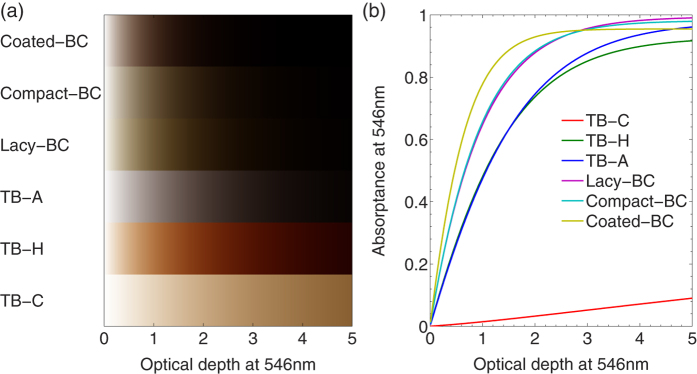
(**a**) Colors of carbonaceous aerosols with realistic particle size distributions as a function of optical depth at the wavelength of 546 nm. (**b**) Absorptance of the aerosol layer at the wavelength of 546 nm.

Although this study determines the colors of ambient aerosols by their relative transmittances at different wavelengths, they nevertheless become blackish if the layer absorptions become large enough. Figure 5 roughly illustrates the relationship between carbonaceous aerosol color and the absorptance. Figure 5a shows the color variations of six different carbonaceous aerosols in the atmosphere at different optical depths, i.e. aerosol amount. Particles with realistic size distributions and geometries are considered for the simulations, which are those given in Table 1. Obviously, different colors are shown for different carbonaceous aerosols and those at different stages, and the brownish colors displayed in Fig. 5a are quite similar to those of the realistic smoke given in Fig. 1a. Figure 5b illustrates the absorptance of aerosol layer as a function of optical depth, and the absorptance denotes the ratio of the flux absorbed by the aerosol layer to the incident flux. The TB-C has much smaller absorptances than the other five cases, which is expected, and the BCs have stronger absorption than the TBs. Again, the Lacy- and Compact-BC give very similar results, because the lacy and compact aggregates have quite close optical properties, especially the absorption cross section^19^,^20^,^21^. However, the Coated-BC absorbs more energy than the BC aggregates at optical depth less than 3, and the absorption becomes saturated at thicker aerosol layers. When the optical depth becomes larger than approximately 3, the absorptance of the Coated-BC is smaller than those of the BC aggregates. This is because of that the coating enhances both the absorption and scattering of BC particles^22^,^23^,^24^, and more energy are reflected before being absorbed by the Coated-BC. From Fig. 5, we conclude that carbonaceous aerosols, either BC or TB, become blackish as the absorptance at 546 nm becomes larger than approximately 0.8.

### Colors of externally mixed biomass burning aerosols

The biomass burning aerosols in the atmosphere always exist in the form of mixtures with different components such as purely-scattering OA, BrC, and BC, and we also investigate the effects of aerosol mixing on their colors. We consider the external mixtures of purely-scattering OA (referred to as Scattering-OA) and each of the six absorptive carbonaceous aerosols considered above. The Scattering-OA is assumed to be spheres, and a constant refractive index of 1.5 is used to obtain their optical properties at the RGB wavelengths (imaginary part is zero)^25^,^26^. To account for the effects of external mixing, the optical properties of the mixture are obtained by averaging those of the Scattering-OA and one kind of the TBs/BCs based on their fractions, and there is no difference on color simulation and display.

**Figure 6:**
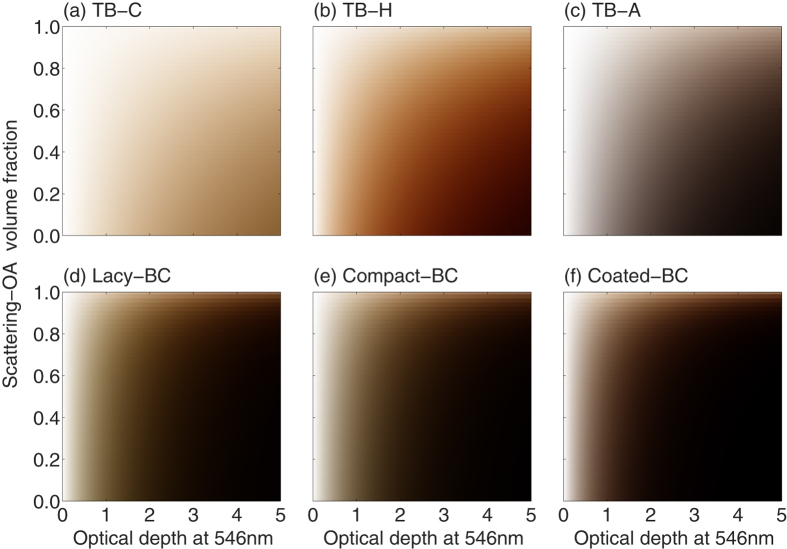
Colors for external mixtures of absorptive and purely-scattering carbonaceous aerosols at different optical depths.

Figure 6 shows the simulated colors of the mixtures. Again, realistic size distributions are used for absorbing carbonaceous aerosols. The y-axis of Fig. 6 is for the volume fraction of the Scattering-OA, and the x-axis is still for the optical depth. It’s easy to understand that the color becomes brighter as the Scattering-OA fraction increases. It should be noticed that the mixtures with the Scattering-OA does not change the AAE of the original absorptive aerosols, whereas the EAE differs. Again, for the TBs, their mixtures with the Scattering-OA show clearly brownish color, and the color becomes darker as the TB-C or TB-H fraction and optical depth increase. The TB-A and BC all show grey or black colors, whereas differences are noticeable for BCs in different formats. It should be noticed that, for the BCs with three different geometries, the BC amount is fixed in the figure. At realistic size distributions, the BCs look more blackish than the TB-H, but still exhibit some brownish color, whereas the TB-A, which has larger size and small AAE, displays almost no brown color.

## Discussion

We investigate the colors of ambient biomass burning aerosols based on the light scattering, radiative transfer and RGB color models. Various carbonaceous aerosol types along with their mixtures of non-absorptive particles are considered, because actual biomass burning smokes are mixtures of various carbonaceous aerosols and minor scattering aerosols. The presented color simulation is, to our knowledge, the first quantitative study on the color of the ambient biomass burning aerosols.

Our simulations have revealed that all carbonaceous aerosols look brownish at small particle sizes and blackish at large ones, because the aerosol AAE largely controls the color and larger particles produce smaller AAE values. At the realistic particle size distributions, BCs, irrespective of their geometry, are shown to be more blackish than tar balls, and the influence of BC aging and non-absorptive coating (i.e. sulfate in our study) on its color in the atmosphere is found to be insignificant. When the absorptance of the layer becomes larger than approximately 0.8, all these carbonaceous particles become blackish. Therefore, our study undermines a common thinking that the blackish color of biomass burning smoke is simply due to BC. Blackish color comes from dense smoke due to either high BC concentration or high TB concentration. In the aspect of how blackish than brownish particles are, we have shown here that some tar balls (e.g. TB-A^8^) appear actually more blackish than BC. Furthermore, with the color simulations here, we demonstrate that the color of biomass burning aerosols can be used to roughly understand the amount of BC+BrC and the ratio of BC to BrC, and to guess the composition of biomass burning aerosols. However, there is still a long way to analyse the smoke color quantitatively based on the current study.

Although the colors we obtained looks quite realistic, the simulations in the present study are highly simplified. Factors such as gas absorption and scattering, spectral response functions of human eyes, anisotropic transmission and three-dimensional effects should be accounted in order to display more accurate aerosol colors, and will hopefully be addressed in follow-up studies. Furthermore, the uncertainties on the refractive indices of carbonaceous aerosols and their influences on the color simulation should also be noticed and investigated in the future.

## Methods

### Biomass burning aerosols

The color of aerosols in the ambient atmosphere is determined by their spectral-dependent optical properties, which are affected by their material (i.e. refractive index), shape and size distribution. This study considers the biomass burning aerosols, and some typical carbonaceous aerosol species as well as their mixtures are discussed. Generally, the biomass burning aerosols we considered can be classified into three types as follows.

The first species considered is tar balls (TBs) analysed by Chakrabarty *et al.*^6^, which we refer to as “TB-C”. Although they claimed that they had generated TBs, in reality they generated particles from smoldering combustion of specific woods and attempted to remove the BC influence from these particles, and to characterize the remaining particles. These remaining particles, which we refer to as TB-C following Chakrabarty *et al.*, are actually a mixture of many OA species rather than a single TB species. Meanwhile, Hoffer *et al.*^7^ did generate TBs in a laboratory experiment, and measured their optical properties. The measured optical properties radically differ from those identified by Chakrabarty *et al.*, which means that Chakrabarty *et al.* might simply analyze a mixture of organic aerosol species from smoldering biomass burning combustion. Furthermore, Chakrabarty *et al.*’s OAs exhibit the sunlight absorption characteristics similar to the BrCs analyzed by Kirchstetter *et al.*^3^.

The second type we address is real TBs, a class of BrC, which are much more absorptive than the TB-C mentioned above. TBs are widespread in biomass burning smoke^6^,^7^,^8^,^9^,^10^,^11^,^27^,^28^, and are typically present as solid spherical particles and can be readily identified by transmission electron microscopy. The TBs generated by Hoffer *et al.*^7^ are relatively fresh TBs, and we refer to them as “TB-H”. The BrC particles discovered by Alexander *et al.*^8^ are likely TBs as well, in which they have very similar absorptivity (i.e. imaginary part for refractive index) to that of the TB-H. Jacobson^29^ took a view that the BrC particles observed by Alexander *et al.* are TBs. However, the TBs studies by Alexander *et al.* show somewhat different spectral variance of absorption than those generated by Hoffer *et al.*^7^. One plausible explanation for this difference is that Alexander *et al.*’s particles are greatly aged TBs, since they found them over the Yellow Sea, where aerosols from China are transported eastwards. In view of this, we refer to Alexander *et al.*’s TB as “TB-A”.

The last species we address is BC, and particles with the same species but different geometries are considered. When BC is just formed, these fresh BC particles are normally in the form of lacy aggregates containing numerous small spheres (diameter of a few tens nanometers)^30^. During the aging processes, the BC clusters may fold up to be more spherical and compact^30^,^31^. The optical properties of realistic cluster are significantly different from those of spherical particles^19^,^32^. Thus, we name the BC with lacy cluster structure as “Lacy-BC”, and those with compact geometry as “Compact-BC”. Furthermore, the BC can easily be coated by other scattering materials such as sulfate and OA during aging process, and a core-shell structure is assumed to account for the “Coated-BC”^22^,^33^,^34^. In this study, we assume the coating and the BC core to have the same volume, and the coating is assumed to be sulfate, the refractive indices of which is obtained from Palmer and Williams^35^. The Lacy-BC is understood as fresh BC, whereas the Compact- and Coated-BCs can be interpreted as aged ones. For the Lacy-, Compact- and Coated-BCs, the BC component (i.e. corresponding refractive index) and volume are kept the same, and we consider the change due to particle geometries, i.e. aging and coating processes.

### Color simulation

We choose to present “color” numerically based on a RGB color model^36^, in which three additive primary colors, i.e. red, green and blue, are added together to reproduce a broad array of colors. The RGB color model is based on the Young-Helmholtz theory of trichromatic color vision^37^, and is widely used for sensing, representation, and display of images in electronic systems. For numerical representations, a color in the RGB color model is determined by quantifying how much each of the red, green and blue is included. Thus, the color can be expressed as a RGB triplet (r, g, b), each component of which may vary from zero to a defined maximum value. Values between 0 and 1 are used to represent each colorant in this study. For instance, (0, 0, 0) represents black, and (1, 0, 0), (0, 1, 0) and (0, 0, 1) correspond to the red, green and blue components, respectively. The red, green and blue monochromatic primary colors are chosen to have wavelengths of 700 nm, 546 nm and 436 nm in this study^38^.

Based on the RGB color model, three steps are needed to obtain the color of ambient aerosols. First, the bulk scattering properties of an ensemble of aerosol particles with given size distribution are calculated, and, to be more specific, the extinction efficiency, single-scattering albedo and asymmetry factor at the red, green and blue wavelengths are estimated^39^. We use the Lorenz-Mie^17^, multiple-sphere T-matrix^14^ and core-shell Mie theories^18^ to calculate the single-scattering properties for spheres, aggregates and core-shell spheres, respectively, and numerical integrations are carried out to give the bulk scattering properties^19^. Second, the multiple scattering and absorption of a homogeneous aerosol layer is accounted for by using a radiative transfer model independently at the red, green and blue wavelengths. A two-stream radiative transfer model is used, and it can efficiently simulate the reflectance, transmittance and absorptance of an aerosol layer (i.e. the ratio of reflected, transmitted and absorbed flux to the incident flux)^40^. Third, we assume that a completely white light with the RGB triplet of (1, 1, 1) goes through the aerosol layer, and the transmitted light is used in the RGB color model to display the color of the aerosol layer. Values of the transmittances obtained from the two-stream radiative transfer model at the red, green and blue wavelengths are assigned to a RGB triplet, i.e. (*T*_R_, *T*_G_, *T*_B_), and then the color can be simply displayed. Figure 1b illustrates the circumstance for our simulations, i.e. incident light passing through an aerosol layer and reaching an observer at the bottom of the layer.

In the numerical simulation, a few assumptions are made to simplify the radiative transfer and color simulation. First, we are interested in the cases with relatively large aerosol optical depth, and the absorption and scattering by gas molecules are ignored. Second, a plane-parallel model with a single homogeneous aerosol layer is considered, which means that we do not consider the complicated spatial variance of smoke density and the three-dimensional radiative transfer effects. Third, the simplest two-stream approximation^40^ is used to account for the reflected and transmitted energy, because it provides approximated isotropic reflected and transmitted radiation and simplifies the complicity on source and viewing geometries. Furthermore, the two-stream model is able to provide reasonable results for a wide range of optical depth and incident angles. The application of the two-stream approximation can be traced back to the phenomenological reasoning regarding the transfer of radiation in a foggy atmosphere given by Schuster^41^, and the details of the two-stream formulations can be found in^40^. Even with more sophisticated and more accurate radiative transfer models available, this study uses the simple assumptions and methods, because we try to focus on the interested variables, i.e. the biomass burning aerosols of different species and their optical properties. Furthermore, the current model includes the fundamental processes for displaying smoke colors, and is simple and efficient enough to be carried out for large amount of simulations.

## Additional Information

**How to cite this article**: Liu, C. *et al.* The colors of biomass burning aerosols in the atmosphere. *Sci. Rep.* **6**, 28267; doi: 10.1038/srep28267 (2016).

## Figures and Tables

**Table 1:** Microphysical and optical properties of the carbonaceous species.

**Type**	**Absorptivity**	**Geometry**	**Diameter**^a^ **(nm)**	**EAE**^b^	**AAE**^c^
TB-C	Weak	Sphere	80	1.6	4.3
TB-H	Moderate	Sphere	100	2.6	2.8
TB-A	Moderate	Sphere	230	0.50	0.62
Lacy-BC	Strong	Lacy aggregate	120	1.4	1.2
Compact-BC	Strong	Compact aggregate	120	1.2	1.0
Coated-BC	Strong	Core-shell sphere	120	1.1	0.92

^a^The diameter is referred to as the particle geometric mean diameter of the lognormal size distribution, and the values close to the observed values are used following^6^,^7^,^8^. For Coated-BC, the diameter is that of the BC core, and the volume of the BC core and sulfate shell is assumed to be the same for each particle.

^b^EAE means the extinction Ångström exponent.

^c^AAE means the absorption Ångström exponent.
